# Artisanal tannery wastewater: quantity and characteristics

**DOI:** 10.1016/j.heliyon.2021.e08680

**Published:** 2021-12-25

**Authors:** Miriam Appiah-Brempong, Helen Michelle Korkor Essandoh, Nana Yaw Asiedu, Samuel Kwame Dadzie, Francis Warings Yao Momade

**Affiliations:** a Regional Water and Environmental Sanitation Centre Kumasi, Department of Civil Engineering, College of Engineering, Kwame Nkrumah University of Science and Technology, Kumasi, Ghana; b Department of Chemical Engineering, College of Engineering, Kwame Nkrumah University of Science and Technology, Kumasi, Ghana; c Department of Materials Engineering, College of Engineering, Kwame Nkrumah University of Science and Technology, Kumasi, Ghana

**Keywords:** Physicochemical parameters, Microbes, Water use, Wastewater, Leather, Ghana

## Abstract

Tannery wastewater is one of the most toxic waste generated in industries. In spite of this, there still remains a paucity of information on characteristics of wastewater generated from artisanal tanneries. This study, therefore, assessed the water consumption, wastewater generation rates, physicochemical and microbiological characteristics of wastewater produced from each process unit of an artisanal tannery in Ghana. The study revealed that the total amount of water use in the tannery ranged between 1171 and 2120L/day whilst the total volume of wastewater generated was within 820 and 1324L/day. Physicochemical characteristics of the different wastewater types generated at the tannery including chemical oxygen demand (13600–24333.30 mg/L), biochemical oxygen demand (1445.64–2803 mg/L), ammonia (3.20–21.38 mg/L), colour (950.35–53900.10PtCo), electrical conductivity (8170 - 10080 μS/cm), turbidity (450.24–1805NTU), suspended (1033.50–3216.40 mg/L) and dissolved (26166.50–4996.65 mg/L) solids exceeded the guidelines set by the Ghana Environmental Protection Agency. There were also high levels of chlorides, sodium, sulphates and calcium ions. The most dominant anion and cation in the wastewater were chlorides (715–20490.60 mg/L) and sodium ions (258–14056.45 mg/L) respectively. Heavy metals identified in the wastewater included zinc, aluminium, iron and chromium ions with the most dominant one being aluminium ions (0.58–78.18 mg/L). Whilst the *E-coli* was below detectable limit, the count of total coliforms ranged between 0 and 4.5 × 10^4^CFU/100mL. Five helminth egg species (*Ascaris lumbricoides,* hookworm*, Trichuris trichiura*, *Strongyloides stercoralis,* and *Enterobius vermicularis*) were identified with their numbers surpassing the safe limit set by the World Health Organisation for irrigation purposes. These results indicated that the indiscriminate discharge of the untreated wastewater on the bare soil as it is practised at the tannery has the potential to adversely affect public and environmental health. Appropriate treatment schemes are therefore, required to treat the wastewater to safe limits prior to discharge.

## 1 Introduction

Water is an essential resource for manufacturing industries. The average water consumption by industries is estimated at 22% of the global water use (Gutterres et al., 2015) with the highest consuming sectors being the textile, semiconductor and leather manufacturing industries (UNIDO, 2007). Industrial use of this important commodity leads to the generation of large volumes of wastewater accompanied with its discharge and treatment issues.

The leather manufacturing sector makes non-putrescible leathers from biodegradable skins and hides via tanning processes (Dargo and Ayalew, 2014). From ancient times, artisanal tanners have relied on tannins; extracts from plant parts such as such as barks, fruit pods, leaves and roots as tanning agents in converting skins/hides into leathers in a process termed as vegetable tanning (Falcão and Araújo, 2018). This artisanal method of leather production is usually carried out using very simple tools in pits and pots in the open space (Ezenwe et al., 2001).

The leather manufacturing process has, however, been heavily industrialised over the years. In modern times, leather making processes are executed in well-built factories equipped with sophisticated machineries and rely on the use of diverse process chemicals (Ezenwe et al., 2001). Consequently, the vegetable tanning method has been substituted with other tanning techniques such as chrome, alum, aldehyde, zirconium salt and syntans tanning (Covington, 1997). Presently, the chrome tanning technique which uses basic chromium sulphate as its tanning agent is practised in about 90% of all tanneries worldwide due to its apparent advantage over the vegetable tanning method in producing very flexible, colourful and multipurpose leathers within a shorter period of time (Dargo and Ayalew, 2014). Despite this enormous transformation in the leather industry, the artisanal method of leather production is still being practised in some developing countries such as Ghana (Ezenwe et al., 2001).

Leather industries, in spite of the attractive leathers produced have been stigmatised with odoriferous environs and generation of highly decomposable organic wastes (Muthukkauppan and Parthiban, 2018). This challenge stems up from the use of animal skin/hides and the dependence on various process chemicals which include dyes, salts, tannins, oils, lime, biocides, enzymes, chromium sulphates, acids and finishing solvents applied in the manufacturing processes (Ilou et al., 2014). Only a small portion of about 20% of most of these chemicals are retained in the leathers during production, the remaining amount comes out with the resulting wastewater (Muthukkauppan and Parthiban, 2018). About 40% of the chromium used in industrial tanneries forms part of the wastewater generated. Chromium in the wastewater can exist in different oxidation states-either as hexavalent chromium [Cr(VI)] or trivalent chromium [Cr(III)] with the former being about a hundred times more toxic than the latter (Genawi et al., 2020). The wastewater generated in tanneries is therefore, generally characterized by strong colouration, huge loads of suspended particles, organic compounds, heavy metals as well as odorous smells (Ilou et al., 2014). In a study conducted by Liu et al. (2002), tannery wastewater ranked among the most toxic industrial wastewaters.

Despite its high toxicity, tannery wastewater are usually discarded on the tannery sites or into nearby surface water bodies without treatment (Amanial, 2016; Chowdhury et al., 2013), particularly in some developing countries such as Ghana leading to environmental degradation. Appropriate wastewater treatment and management schemes are required to curb this menace. Information regarding water consumption, wastewater generation volumes and its characteristics are required for the design and management of these treatment schemes and also for the environmental impact assessment of indiscriminate discharge of tannery wastewater. Whilst this information is readily available for industrial tanneries (Sawalha et al., 2019; Ilou et al., 2014; Islam et al., 2014), there is a paucity of it on artisanal tanneries, even though artisanal leather making remains a source of livelihood for lots of people in Ghana and in some other developing countries such as Ethiopia (Gebremichael, 2016), Cameroun (Paltahe et al., 2019), Sudan (Skinner, 2007) and Nigeria (Zaruwa and Kwaghe, 2014). From an extensive literature search, a study conducted by Paltahe et al. (2019) was the only work done investigating into the characteristics of artisanal tannery wastewater. This paper therefore, seeks to fill this gap in literature by investigating the water consumption, wastewater generation rate and characteristics in an artisanal tannery in Ghana.

### 1.1 General processes in artisanal leather production in Ghana

Skins of sheep and goats usually obtained from slaughterhouses are cured by salting and sun-drying to prevent decay during storage. The leather making process as practised in the selected study area starts with the liming process where the cured skins are soaked in a liming liquor prepared from a mixture of wood ash, waste carbide and water in order to loosen the hair roots in the skins. The hairs are then removed from the skins using a double-handled knife. Next, the skins are drenched in a deliming and bating solution made from a mixture of ground pawpaw leaves and water. The proteolytic enzyme, papain in the pawpaw leaves disintegrates the flesh and the fatty tissues on the skins which are then scraped off with the knife. The skins after been bated are washed and then tanned via the vegetable tanning technique. The vegetable tanning liquor is composed of ground pods of *Acacia nilotica* (commonly termed as Gum Arabic tree or Babul) and water. The tanned skins may then be dyed into red or black colour. The red dye liquor is produced from a mixture of pounded sorghum leaf sheaths, a small portion of the liming liquor and water. The black dye on the other hand, is composed of water, iron filings and ground pods of *Acacia nilotica.* The leathers after being dyed are dried, stretched and trimmed to complete the leather manufacturing process. On very rare occasions, the tanners produce white leathers by employing the use of aluminium sulphate in tanning the skins-a process termed as alum tawing. The alum tawing liquor is a mixture of aluminium sulphate, salt and water.

**Figure 1:**
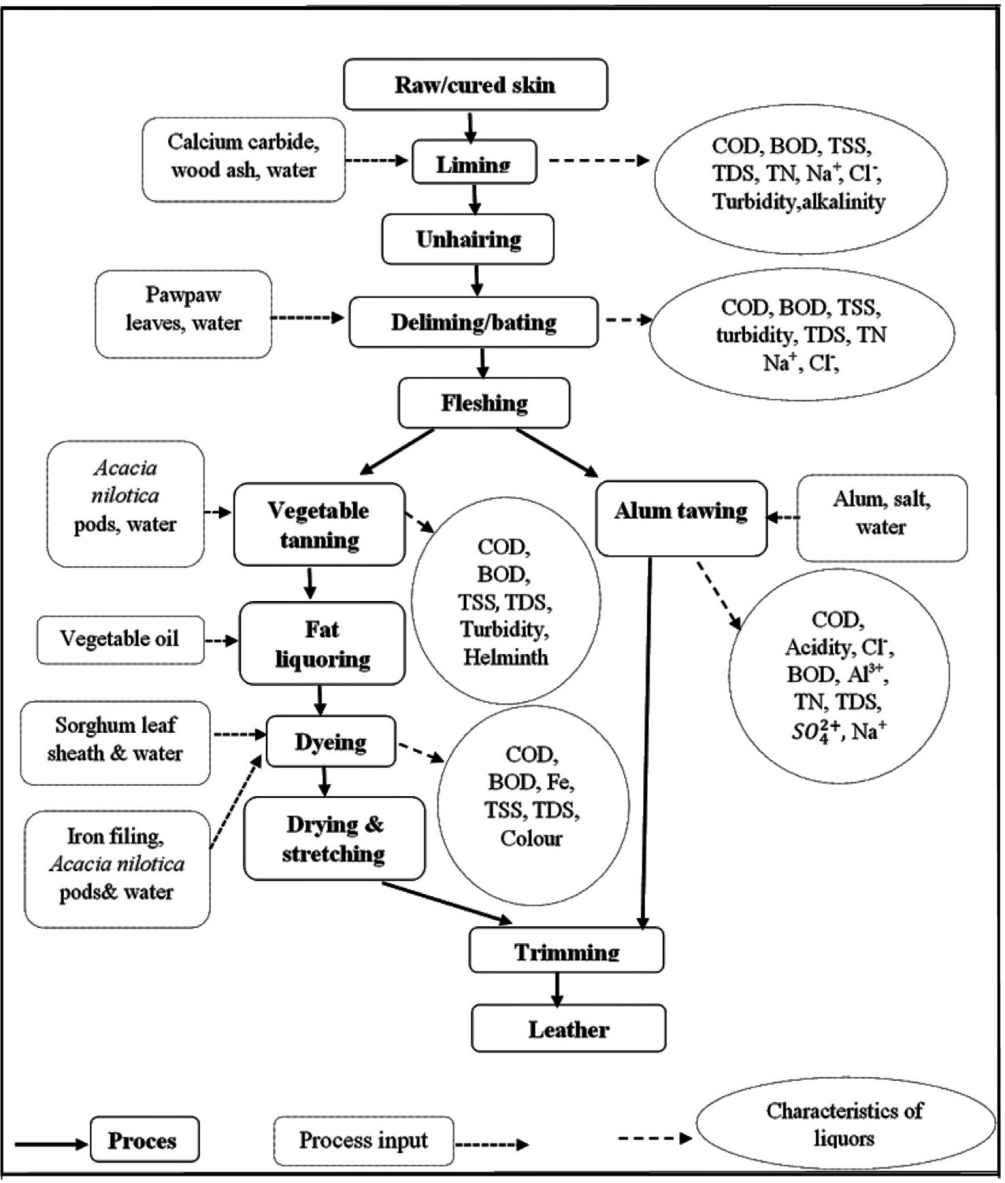
Flow chart of leather making process in an artisanal tannery with its input materials and characteristics of the tannery liquors.

Amongst all the different liquors employed at the tannery, namely, liming, deliming/bating, vegetable tanning, alum tawing, red dye and black dye liquors, only the alum tawing, vegetable tanning and red dye liquors are discarded after use. The remaining liquors are reused repeatedly in the production process and frequently strengthened again by adding the appropriate process material or chemical when their potency diminishes. A schematic diagram illustrating the production processes and the input materials used in artisanal leather making in Ghana has been shown in Figure 1. Detailed descriptions on the leather making processes in artisanal tanneries in Ghana are well elaborated in a study conducted by Appiah-Brempong et al. (2020).

## 2 Materials and methods

### 2.1 Data collection

The data was collected from the Aboabo Artisanal Tannery in Ghana with a geographical location of 6^o^41′50.57``N, 1^o^36′7.02``W. Data gathering encompassed field data collection and laboratory analyses. The field data collection was employed in determining the water demand and wastewater generation volumes as well as in the sample collection of process liquors. The laboratory analyses were then conducted to determine the types and levels of pollutants in the process liquors.

#### 2.1.1 Quantification of water demand and wastewater generation

Water demand and wastewater generation in leather industries are generally measured in L/kg of skin or hide. Hence, to estimate the water consumption and wastewater generation volume during each production stage, the average weight of the animal skins to be processed as well as the volumes of water used and the wastewater produced afterward are required. The production stages in artisanal leather making which involves the use of water are liming, deliming & bating, vegetable tanning, alum tawing, red dyeing, black dyeing and washing. Due to the lack of a water metering system at the tannery the water use and wastewater volumes were measured in litres using graduated buckets. In this way, the total volume of water used in each process unit could be read and computed. The leather making operations were carried out in plastic buckets instead of the pits in order to enhance accuracy in the measurements. The spent liquor obtained after the vegetable tanning, alum tawing and red dyeing operations were carefully poured out individually into specified graduated buckets to determine the volume of wastewater generated in each operation stage. Prior to the commencement of the leather manufacturing operations, the mass of the raw animal skins were weighed in kilograms using a hanging scale. The water demand and volume of wastewater generated (L/kg) at each process stage were computed by dividing the total volume of water used or wastewater generated (L) by the total mass of raw animal skins (kg) that were processed [Eqs. (1) and (2)]. The quantification process was carried out five times on the field over a period of five weeks since the manufacturing process takes 5 days to complete. Data on the number of skins processed daily at tannery were also collected over this 5-week period. This data was used in estimating the daily volumetric water consumption and wastewater generation for each process unit [Eqs. (3) and (4)].

(1) $$V_{w}=\frac{v_{w}}{m_{total,\;skins}}$$

(2) $$V_{ww}=\frac{v_{ww}}{m_{total,\;skins}}$$

where, *V*_*w*_ and *V*_*ww*_ are the average volume of water use and the average volume of wastewater generated respectively in each process stage per unit mass of raw animal skin (L/kg), *v*_*w*_ and *v*_*ww*_ are the total volume of water used or wastewater generated respectively in each process unit (L) and *m*_*total, skins*_ is the total mass of raw skins to be processed (kg)

(3) $$q_{d,w\;}=\;V_{w}\times N\times m_{average,\;skins}$$

(4) $$q_{d,ww\;}=\;V_{ww}\times N\times m_{average,\;skins}$$

where, *q*_*d,w*_ and *q*_*d,ww*_ are the average daily volumetric water use and average daily volumetric wastewater generated (L/day) respectively, *N* is the average number of skins processed in a day (1/day) and *m*_*average, skins*_ is the average mass of a raw animal skin to be processed (kg).

#### 2.1.2 Wastewater sample collection and characterisation

Grab samples of the different liquors/wastewater (liming, deliming/bating, black dye, vegetable tanning, alum tawing and red dye liquors) meant for physicochemical and microbial analyses were collected from their respective receptacles at the tannery into twelve (12) 1L plastic bottles for laboratory analyses. Each sample type was collected in duplicates. Samples designated for heavy metal analyses were acidified to pH ranges between 1 and 2 using nitric acid in order to reduce the sorption of heavy metals onto the walls of bottles and also to prevent the formation of metal precipitates during sample storage. Parameters which can alter easily such as temperature, pH, turbidity and electrical conductivity were measured on site. Sample bottles were transported to the Environmental Quality Engineering Laboratory of Kwame Nkrumah University of Science and Technology in an ice chest containing ice cubes. Sample collection was done on five different days in the month of May, 2019. Samples which were not analysed immediately were stored at 4 °C in a refrigerator to hamper microbial activities. All analyses were done in accordance with standard methods (American Public Health Association (APHA), 1999). Turbidity and Total Suspended Solids (TSS) measurements were done using the HANNA turbidimeter (HI 93414) and the gravimetric method respectively. The HACH methods for determination of Chemical Oxygen Demand (COD), sulphate (${\text{SO}}_{4}^{2+}),\;$ ammonia (NH_3_–N), and Total Nitrogen (TN) levels in the liquors were followed and the results were read using HACH DR 3900 spectrophotometer. Biochemical Oxygen Demand (BOD_5_) analysis was carried using the dilution method alongside with dissolved oxygen measurement. Atomic absorption spectroscopy was employed in analyzing calcium (Ca^2+^), sodium (Na^+^), aluminium (Al^3+^), zinc (Zn^2+^), chromium (Cr) and iron (Fe). Chloride (Cl^−^) was measured using the argentometric method. The determination of total coliforms and *E-coli* were performed using the membrane filtration method with Chromocult® coliform agar and detection and enumeration of helminth eggs carried out according to the modified USEPA technique (Schwartzbrod, 1998).

## 3 Results and discussion

### 3.1 Water use and wastewater generation at the artisanal tannery

A total amount of 10–18L of water is used on each kilogram of raw animal skin during leather production at the artisanal tannery. The corresponding wastewater volume generated ranged from 7 to 11.30L of wastewater per kilogram of raw skin. The number of raw skins processed daily was averagely 57 and the average weight of a skin was also found to be 2.06kg. Hence, the daily water consumption and wastewater generation range from 1,170 to 2,120L and 820–1320L respectively. The water consumption and the wastewater generation rate for each production unit of the artisanal tannery in L/kg raw skin and in L/day are presented in Table 1.

**Table 1:** Water consumption and wastewater generation volumes during each production stage in an artisanal tannery.

Process	Water Consumption		Wastewater generation	
	L/kg raw skin	×10^2^ L/day	L/kg raw skin	×10^2^ L/day
**Pre-tanning**				
Liming	1–4	1.17–4.69	0	0
Deliming/Bating	0.5–1.4	0.59–1.64	0	0
**Tanning**				
Vegetable tanning	1.2–1.6	1.41–1.87	0.7–1.3	0.82–1.52
Alum tawing	4–6	4.69–7.03	3.5–6	4.10–7.03
**Post-tanning**				
Black dyeing	0.5–0.7	0.59–0.82	0	0
Red dyeing	0.5–1.0	0.59–1.17	0.5–0.7	0.59–0.82
**Composite washing**	2.3–3.4	2.69–3.98	2.3–3.3	2.69–3.87
**Total**	**10.0–18.1**	**11.71–21.20**	**7.0–11.3**	**8.20–13.24**

Water use and wastewater generated at the artisanal tannery is less than the 35–40L of water/kg raw hide and 30–40L of wastewater/kg raw hide widely reported in literature for industrial leather manufacturing industries (Thanikaivelan et al., 2005). Whilst about 65% of the total amount of water consumed in the artisanal tannery is discarded as wastewater, approximately 90% of the water used in industrial tanneries is discharged as wastewater (Kabir et al., 2017). The water consumption in the artisanal tannery is however, close to the 12.7–17.6L/kg raw hide reported by Sundar et al. (2001) for advanced leather manufacturing industries where cleaner technologies aimed at reducing water demand and pollution loads are practised. The wastewater generation rate at the artisanal tannery is also comparable to that of advanced leather manufacturing industries (10–12L/kg) (Sawalha et al., 2019).

The disparity in water demand between artisanal and most industrial tanneries can be explained by the predominant use of bovine hides (Dixit et al., 2015) in industrial leather production industries as opposed to the extensive use of sheep and goat skins in the artisanal tannery. Larger quantities of water are required in processing bovine hides due to the bulkiness of these hides. The weight of the hides is estimated to be within the range of 15 and 40kg whilst that of the sheep and goatskins are within 1 and 6kg (European-commission, 2003).

The few washing stages incorporated in the manufacturing process of artisanal tanneries can also explain its low water use. Whilst the hides are washed after almost every treatment stage primarily to rid them of any absorbed process chemicals in industrial tanneries (Nacheva et al., 2004) this is rarely the case in artisanal tanneries where washing may be done only after bating the pelts or during dyeing when fainter colours are desired. Thus, an amount of 11–13L of water/kg of raw hide is apportioned for washing purposes in industrial tanneries (Sundar et al., 2001) whilst only about 2.3–3.4L of water/kg of raw skin is meant for washing purposes during artisanal leather production. According to Boahin et al. (2013) the inadequate washing of the skins by the artisans contributes to the odourous smell of the finished leathers.

Furthermore, the differences in leather manufacturing processes in industrial and artisanal tanneries could also account for the variation in water demand. Some operations such as pickling (0.8–1L/kg raw hide), rechroming (0.7–1L/kg raw hide) and neutralization (1.7–2L/kg raw hide) which require extensive use of water (Sundar et al., 2001) are practised in industrial tanneries but not in the artisanal tannery. Additionally, the soaking stage, a very important step in leather making aimed at washing and rehydrating the skins is omitted from the production stages in the artisanal tannery but is however, widely practised in industrial tanneries and consumes as much 6–9L/kg of raw hide (Sathiyamoorthy et al., 2013; Sundar et al., 2001). The reuse of the liming, deliming/bating and black dye liquors is a water-saving strategy which could also account for the lesser use of water in the artisanal tannery.

Finally, some amount of water termed as technical water is used in industrial tanneries for energy production, wastewater treatment and floor cleansing (Buljan et al., 2000; Ramanujam et al., 2010) constitutes about a fifth of the total water consumption (Buljan et al., 2000). However, in the artisanal tannery, where leather production takes place manually in an open space with no wastewater purification systems, there is no allocation of water for sanitary purposes or energy production.

Considering the wastewater discharge, the lesser volume generated in the artisanal tannery can be linked to the low amount of water used as well as the reuse of some of the liquors in the leather manufacturing processes as it has been also reported for advanced leather making industries (Sawalha et al., 2019). Additionally, absorption of water by some of the solid process materials such as waste carbide, wood ash and ground pods of *A. nilotica* used in the artisanal leather production process can also account for the lower volumes of wastewater from the artisanal tannery. This forms a sludge at the bottom of the pots or pits. This is however, unlike industrial leather making where according to Ilou et al. (2014), most of the process chemicals used are in a liquid form which rather add to the resulting wastewater since these chemicals are not fully absorbed into the hides or leathers.

### 3.2 Characteristics of the artisanal tannery spent and unspent liquors

The results of the physical, chemical and microbiological characteristics of the spent (vegetable tanning, alum tawing and red dye liquors) and unspent liquors (liming, deliming/bating, black dye liquors) are presented in Tables 2, 3, 4. The characteristics of the different liquors are indicated in the schematic diagram of the leather making process shown in Figure 1.

**Table 2:** Physical characteristics of spent and unspent liquors at the artisanal tannery.

Parameter	Liming	Deliming/Bating	Spent vegetable tanning	Spent alum tawing	Spent red dye	Black dye	Ghana EPA (2016)
Temperature (^o^C)	33.22 ± 2.45	33.22 ± 0.83	32.40 ± 1.01	33.60 ± 1.15	33.50 ± 1.08	30.80 ± 0.56	<3^o^ above ambient
Colour (PtCo)	48983 ± 634.11	17566 ± 679.54	22033 ± 1350.5	950.35 ± 123.3	53900 ± 804.65	16886 ± 900.9	100
EC (μS/cm)	56600 ± 26.28	34700 ± 21.50	10080 ± 77.55	52370 ± 3.42	8170 ± 4.30	6930 ± 1.32	1500
TDS (mg/L)	28500 ± 497.80	17533 ± 580.60	4996.7 ± 383.78	26166 ± 261.63	4123.3 ± 173.62	3576.7 ± 108.62	1000
TSS (mg/L)	6728.7 ± 645.42	8116.8 ± 633.88	3216.4 ± 868.78	1033.5 ± 704.65	1894.1 ± 385.01	2820 ± 165.82	50
Turbidity (NTU)	2953.4 ± 279.24	1733.3 ± 394.98	1805 ± 142.93	450.24 ± 43.98	1700 ± 186.6	943.50 ± 74.52	75

**Table 3:** Chemical characteristics of spent and unspent liquors at the artisanal tannery.

Parameter	Liming	Deliming/Bating	Spent vegetable tanning	Spent alum tawing	Spent red dye	Black dye	Ghana EPA (2016)
pH	12.95 ± 0.28	8.79 ± 0.22	6.02 ± 0.47	4.54 ± 0.83	6.36 ± 0.60	5.75 ± 0.74	6–9
BOD_5_ (mg/L)	2705.3 ± 396.73	2073.4 ± 287.51	2803 ± 308.76	1445.6 ± 641.56	2709 ± 354.12	2068.8 ± 291.41	50
COD (mg/L)	17050 ± 157.20	4366.7 ± 921.65	24333 ± 654.84	2380 ± 109.23	13600 ± 157.55	6970 ± 72.10	250
BOD_5_: COD	0.16 ± 0.06	0.47 ± 0.04	0.12 ± 0.02	0.61 ± 0.06	0.20 ± 0.04	0.30 ± 0.06	
TN (mg/L)	115.68 ± 21.85	93.14 ± 14.45	37.18 ± 9.16	64.35 ± 55.87	38.52 ± 7.02	40.10 ± 36.77	50
NH_3_–N (mg/L)	68.75 ± 7.43	48.08 ± 5.79	16.80 ± 3.38	21.38 ± 3.54	3.20 ± 0.78	4.75 ± 0.49	1
SO_4_^2+^(mg/L)	355.75 ± 30.74	115.38 ± 19.56	30.14 ± 3.20	1190.7 ± 18.72	90.25 ± 3.80	10.42 ± 2.05	250
Ca^2+^ (mg/L)	18.45 ± 5.78	14.80 ± 3.61	7.26 ± 1.15	9.74 ± 2.38	5.72 ± 1.28	3.24 ± 0.26	-
Cl^−^(mg/L)	23280 ± 1636	12516 ± 151.32	751 ± 68.74	20490 ± 1270.64	795.88 ± 82.35	643.30 ± 76.55	-
Na^+^ (mg/L)	20727 ± 503.28	9510 ± 141.35	258.66 ± 36.95	14056 ± 763.75	315.20 ± 72.61	245.28 ± 45.60	-
Total Fe (mg/L)	7.58 ± 2.40	6.05 ± 2.94	2.02 ± 1.45	5.26 ± 1.72	2.95 ± 1.08	18.90 ± 5.92	10
Al^3+^ (mg/L)	5.04 ± 1.02	3.55 ± 0.75	1.28 ± 0.08	78.18 ± 10.52	0.54 ± 0.01	0.95 ± 0.15	-
Total Cr (mg/L)	0.04 ± 0.001	0.04 ± 0.002	0.04 ± 0.002	0.04 ± 0.002	0.03 ± 0.001	0.2 ± 0.01	0.5
Zn^2+^ (mg/L)	3.18 ± 0.14	5.04 ± 0.64	0.64 ± 0.26	1.54 ± 0.12	0.54 ± 0.18	0.4 ± 0.10	10

**Table 4:** Biological characteristics of the spent and unspent liquors at the artisanal tannery.

Microbes	Liming	Deliming/bating	Spent vegetable tanning	Spent alum tawing	Spent red dye	Black dye	Ghana EPA (2016)
**Bacteria (CFU/100mL)**							
Total coliform	2.6 × 10^3^ (0–6x10^3^)	1.7 × 10^4^ (0–4x10^4^)	4.5 × 10^4^ (0–1x10^5^)	9 × 10 (0–2x10^2^)	5.5 × 10^3^ (0–1x10^4^)	2.2 × 10^3^ (0–4x10^3^)	400
*E-coli*	0	0	0	0	0	0	10
**Helminth eggs (eggs/L)**							
*Ascaris lumbricoides*	5.6 (0–16)	1.40 (0–3)	3.8 (2–8)	1.8 (0–4)	2.8 (0–5)	1.2 (0–3)	-
Hook worm	1 (0–2)	0	3.6 (0–7)	0	3.5 (2–5)	1 (0–2)	-
*Trichuris trichiura*	2.3 (0–5)	1 (0–2)	4.2 (0–9)	0	1.4 (0–3)	1.2 (0–3)	-
*Strongyloides stercoralis*	2.2 (2–3)	1 (0–2)	2.6 (0–6)	0	0.8 (0–1)	3.2 (1–7)	-
*Enterobius vermicularis*	3.8 (0–8)	1.8 (0–3)	4.4 (0–10)	0.8 (0–2)	2.6 (2–4)	2.3 (0–5)	-

The range of values are written in brackets.

#### 3.2.1 Physical parameters

From the results displayed in Table 2, the average temperature of all the liquors ranged within 30.80 and 33.60 °C due to the prevailing weather conditions. The temperature of all the liquors fell within the tolerance limit established by the Ghana Environmental Protection Agency (Ghana EPA).

The mean Electrical Conductivity (EC) and Total Dissolved Solids (TDS) of all the liquors ranged within 6930 and 56600 μS/cm and 3576.65 and 28500 mg/L respectively (Table 2). The liming liquor had the highest EC (56600 μS/cm) and TDS (28500 mg/L) concentrations due to the large concentration of sodium, chloride (from the salts applied on the skins), calcium, and magnesium (from the wood ash and waste carbide) ions. These ions are absorbed into the animal skins during the liming stage and diffuse into the other liquors upon immersion of the skins. Hence, their concentrations in the subsequent liquors dwindled along the process stages from the deliming/bating, vegetable tanning, red dyeing to the black dyeing stage. Furthermore, disintegration of soluble proteins on the skins (Madhavi et al., 2011) might also contribute to the dissolved matter content of the liming and deliming/bating liquors. Additional sources of dissolved solids in the deliming/bating, vegetable tanning, black dye liquor and red dye liquors can also be attributed to the use of the crushed plant materials (pawpaw leaves, acacia pods and the leaf sheaths). That of the alum tawing spent liquor stems from the presence of salts and aluminium sulphate (Al_2_(SO_4_)_3_).

The values of the EC and the TDS of all the spent liquors were above the stipulated Ghana EPA standards of 1500 μS/cm and 1000 mg/L respectively. Discharge of such wastewater on bare lands can lead to an increase in electrical conductivity and solute concentration in soils (Al-Jaboobi et al., 2014) which destroys soil structure and impedes soil drainage, nutrient retention, soil aeration and plant development (Maria et al., 2012). It also results in plant stress (Corwin and Yemoto, 2017) and a decrease in the abundance of essential soil fauna (Maria et al., 2012). Infiltration of such wastewater into water bodies tend to make them unpalatable (Sherrard et al., 1987) and also causes low reproduction rate, death, loss of habitat and species extinction in aquatic organisms (Weber-Scannell and Duffy, 2007). Certain domestic animals such livestock and chickens also die ingesting wastewater laden with large concentrations of dissolved solids (Sherrard et al., 1987).

The Total Suspended Solids (TSS), turbidity and colour of the liquors resulted from the presence of organic and inorganic materials as well as the dyes used in the leather making process (Bilotta and Brazier, 2008). Materials such as hair residuals, wood ash, calcium carbide residues and plant materials contributed significantly to the turbidity and suspended matter constituents of the liquors. From Table 2, the average values of TSS, turbidity and colour ranged within 1033.50 and 8116.75 mg/L, 450.24 and 2953.35NTU and 950.35 and 53900PtCo respectively. The liming liquor was the most turbid due to the presence of the hair residues and the partially dissolved wood ash and waste carbide. The bating and deliming liquor had the highest constituent of suspended matter resulting from the crushed pawpaw leaves, disintegrated animal flesh and fat tissues, hair residues and soil particles. The red dye wastewater had the strongest colouration.

The values of TSS, turbidity and colour obtained for the spent liquors were in excess of the limits proposed by Ghana EPA which are 50 mg/L, 75NTU and 100PtCo respectively. The discharge of this wastewater on bare lands as practised at the tannery can cause a restriction in the flow rate of water through the soil as the soil pores can be clogged by the suspended particles (Vinten et al., 1983). For surface water bodies, not only will the aesthetic value be destroyed but also the photosynthetic activity will be reduced due to diminished light intensity. Thus, growth and abundance of primary producers will be impaired which will ultimately affect the food chain. The suspended particles are likely to block air passages in fishes and smother them to death, destroy habitats, eggs and larvae of fishes and also disrupt free movement and search for food by fishes (Bilotta and Brazier, 2008; Ryan, 1991). Eventually, this can lead to loss in biodiversity and population abundance (Kemp et al., 2011) in surface water bodies.

#### 3.2.2 Chemical parameters

The acidity or alkalinity of a wastewater is determined by its pH. From the results shown in Table 3, the liming and the deliming/bating liquors were found to be basic (12.95 and 8.79 respectively) due to the presence of calcium hydroxide (lime). The calcium hydroxide originates from the reaction between wood ash and calcium carbide with water (Sun et al., 2015; Farhad and Mohammadi, 2005). The pH of the deliming/bating liquor is appropriate to decrease the pH of the pelts so as to favour the bating process (Beghetto et al., 2013). The black dye liquor (5.75) and the vegetable tanning wastewater (6.01) are slightly acidic due to the presence of the tannic acids emanating from the acacia pods (Mahdi et al., 2006). Paltahe et al. (2019) reported pH values of 12.70, 9.10 and 7.10 for liming, deliming/bating liquors and vegetable tanning wastewater respectively which are in agreement with that obtained in this study. The red dye wastewater is also slightly acidic (6.36) due to the dye extract from the sorghum leaf sheaths. This pH value is in line with the pH of 6.2 reported by Gumel and Ali (2012). The alum tawing wastewater is highly acidic (4.54) as a result of the production of sulphuric acid from the dissolution of aluminium sulphate ($Al_{2}{\left(SO_{4}\right)}_{3})$ in water (OMRI, 2015). The pH of all the wastewater types except that of alum tawing were within the permissible range of 6–9 as stipulated by Ghana EPA. Direct discharge of the alum tawing wastewater on the ground can make soils acidic (Al-Jaboobi et al., 2014) resulting in the release of toxic ions such as aluminium and manganese. These ions hinder growth in both plants (Rousk et al. 2009) and soil fauna (Lavelle et al. 1995) and also hampers the absorption of water and essential nutrients by plant's roots (Thakuria et al., 2016; Steiner et al., 2012). Additionally, the acidic wastewater can lead to acidification of groundwater bodies particularly shallow wells which is a major source of water for the residents around the tannery. Acidified groundwater is characterized by a decline in pH and alkalinity and this also stimulates the release of toxic chemicals such as nitrate, aluminium, zinc, cadmium and chromium into the water (Knutsson, 1994; Soveri, 1992).

The average Biochemical Oxygen Demand (BOD_5_) and Chemical Oxygen Demand (COD) recorded in all the liquors occurred within the ranges of 1445.64 and 2803 mg/L and 2380 and 24333.30 mg/L respectively. The organic matter in the liquors stemmed from the use of plant materials (acacia pods, sorghum leaf sheath, pawpaw leaves), oils, as well as the hair, wool, flesh and adipose tissues which detached from the animal skins. Reported values in literature are 7100mgBOD_5_/L and 18330mgCOD/L for liming liquor; 3119mgBOD_5_/L and 8560mgCOD/L for deliming/bating liquor and 3042mgBOD_5_/L and 7640mgCOD/L for vegetable tanning wastewater (Paltahe et al., 2019). Even though these are results from an artisanal tannery in Cameroun they differ from that obtained in this study possibly due to the variation in the number of skins processed and the quantities of plant materials or chemicals used in the preparation of the liquors as there are no strict measurement guiding the preparation of the liquors. The biodegradability index (BOD_5_: COD) of the liquors are also presented in Table 3. Generally, wastewaters with a BOD_5_:COD <0.4 are characterised as being hardly biodegradable which may be resulting from their toxicity or the presence of recalcitrant compounds (Lofrano et al., 2013; Schrank et al., 2004). From the results obtained all the liquors except the deliming/bating and alum tawing liquors had low biodegradability. The low biodegradability of the vegetable tanning, red dye and black dye liquors could be attributed to the phenolic compounds which are the main constituents of the acacia pods and sorghum leaf sheaths (Elgailani and Ishak, 2016; Kayodé et al., 2011; He et al., 2007). Phenolic compounds are not easily biodegradable and are also toxic to microbial organisms (Benson et al., 2013; Doughari, 2012; Schrank et al., 2004). The low biodegradability of the liming liquor can be linked to its high pH (12.95) which could have hampered activities of the microorganisms. The BOD_5_ and COD concentrations of all the wastewater discharges are above the permissible threshold of 50 and 250 mg/L respectively. Discharge of wastewater with high organic loads into receiving water bodies can result in rapid growth of microbes, dissolved oxygen depletion, death of the aquatic organisms, turbid water, and low photosynthetic activities. The decay of the dead organisms produce foul smells in the water bodies and increase the growth of pathogenic organisms (Akpor and Muchie, 2011).

The average total nitrogen (TN) and ammonia (NH_3_–N) concentrations in the liquors were within 37.18 and 115.68 mg/L and 3.20 and 68.75 mg/L respectively (Table 3). The highest amount was found in the liming liquor due to the presence of blood, flesh, soluble proteins (keratin, globulins, albumins, elastin and cells), hairs, fatty tissues and dung (Mittal, 2004). The plant materials used (acacia pods, pawpaw leaves and sorghum leaf sheath) also contain some amount of crude proteins (Abbasian et al., 2015; Nwofia et al., 2012; Adetuyi et al., 2007) which could add to the nitrogenous composition of the various liquors. The concentrations of TN in the alum tawing wastewater and that of NH_3_–N in all the spent liquors were not consistent with the Ghana EPA guidelines (50mgTN/L and 1mgNH_3_/L). The occurrence of flesh and fatty tissues as well as hair residues in the alum tawing liquor could have contributed to its high TN and NH_3_–N concentrations. For the vegetable tanning and red dye wastewaters, the high levels of NH_3_–N could be primarily linked to the presence of plant materials, residues of hair, flesh and adipose tissues. Introduction of excessive amounts of nitrogenous substances in surface water bodies can lead to algae growth, depletion of dissolved oxygen and ultimate destruction of the water quality (Akpor and Muchie, 2011). High levels of ammonia has a toxic effect on fishes by causing growth retardation, tissue erosion, and mortality (Shin et al., 2016).

Sodium (Na^+^) and chloride (Cl^−^) ions were the dominating cations and anions in the wastewater respectively. The mean concentrations of Na^+^ and Cl^−^ ions in the liquors were from 245.28-20727.40 mg/L and 643.30–23280.15 mg/L respectively (Table 3). The liming liquor had the highest constituent of both Na^+^ and Cl^−^ ions since it receives the bulk of the salts applied in preserving the skins. Due to inappropriate washing of the skins, some of the ions retained in the skins diffuse into the subsequent liquors. The sodium levels in the vegetable tanning, red dye and black dye liquors may also be originating from the trace concentrations in the plant material used (Abbasian et al., 2015; Adetuyi et al., 2007). The addition of salt in the alum tawing liquor could also explain its high levels of chloride (20490.60 mg/L) and sodium (14056.45 mg/L) ions. There are no set limits for sodium and chloride ions in the Ghana EPA guidelines for wastewater discharge. Sodium and chloride ions are essential for metabolic processes in plant and animal cells. However, excessive amount of salt have been associated with cardiovascular and renal illnesses in man (Kumar and Puri, 2012), increase in electrical conductivity and salinity in water bodies and soils, corrosion of metallic components of wastewater treatment systems (Kumar and Puri, 2012), osmotic imbalance in fishes impeding growth and reproduction processes (Fontenot et al., 2013), hindrance in seed germination, plant growth, reproduction and absorption of water and essential nutrients in plants (Akbarimoghaddam et al., 2011).

The average levels of sulphate $\left({\text{SO}}_{4}^{2+}\right)$ and calcium (Ca^2+^) ions fell within the ranges of 1190.70 and 10.42 mg/L and 18.45 and 3.24 mg/L respectively (Table 3). The alum tawing wastewater had the highest concentrations of sulphate whilst the calcium ions were mostly predominant in the liming liquor. The least concentrations of the ions occurred in the black dye liquor. The sulphate and calcium ions in the liquors could have stemmed from the build-up of blood and fleshly tissues from the animal skins (Blinn et al., 2006; Ortolani et al., 2001), disintegrated hairs/wool (Reis et al., 1967; Fletcher et al., 1963), animal dung (Saviozzi et al., 2006) trapped in the hairs on the skins as well as the wood ash (Pitman, 2006) added to the liquor. The pawpaw leaves (Glazer and Smith, 1965), acacia pods (Abbasian et al., 2015), sorghum leaf sheath (Adetuyi et al., 2007) and vegetable oils (He et al., 2009) also contain traces of sulphur and calcium ions which could be released into the respective liquors. The calcium carbide is also a good source of calcium ions (Serafimova et al., 2011). Even though there are no set guidelines for Ca^2+^ ions in the Ghana EPA standards, increased levels may lead to increased risk of cardiovascular diseases and kidney stones in man (Daly and Ebeling, 2010). In water bodies, calcium ions can combine with carbonate ions resulting in water hardness which causes corrosion and scaling in water distribution systems and boilers (WHO, 2011). With regards to sulphate ions, only the alum tawing wastewater had concentrations above the Ghana EPA guidelines (250 mg/L). Elevated concentrations of the ions can cause laxative effect, diarrhoea (Bashir et al., 2012) and dehydration in man, alter the taste of drinking water, cause corrosion and scaling in water systems (WHO, 2004) and lead to eutrophication in surface water bodies (Lamers et al., 2002). Sulphates can also be reduced to hydrogen sulphide in water which is very toxic to living organisms (WHO, 2003). High levels of sulphate in soils tend to enhance the growth of sulphate-reducing bacteria which are responsible for the production of methyl mercury, a very toxic compound which can lead to brain malfunctioning and death in animals and man (Jeremiason et al., 2006).

The different heavy metals analysed were total iron, aluminium, zinc and total chromium. The concentrations found in the different liquors varied between 2.02 and 18.90mgFe/L, 0.54 and 78.18mgAl^3+^/L, 0.4 and 5.04mgZn^2+^/L and 0.03 and 0.2mgCr/L (Table 3). The black dye liquor had the highest concentration of total iron, chromium and zinc ions which could be attributed to the use of iron filings in the solution. Other sources of total iron and zinc in the liquors are the animal blood, hair, disintegrated skin proteins (Gupta, 2014; Ganz and Nemeth, 2006; Top, 2005) and wood ash (Karltun et al., 2008). The pawpaw leaves, acacia pods and sorghum leaf sheaths also contain traces of total iron and zinc ions (Singh et al., 2016; Abbasian et al., 2015; Nwofia et al., 2012; Adetuyi et al., 2007). The alum tawing spent liquor had the highest levels of aluminium ions originating from the application of aluminium sulphate in the solution. Wood ash is another source of aluminium ions (Karltun et al., 2008) and pawpaw leaves are also known to contain some concentrations of chromium (Sharma et al., 2013).

The levels of iron, total chromium and zinc ions in the different types of wastewater met the Ghana EPA standard (10mgFe/L, 0.5mgCr/L and 10mgZn/L). There are however, no specified threshold concentrations for Al^3+^ in the Ghana EPA guidelines. Aluminium is a non-essential nutrient in living things (Akpor and Muchie, 2011). Aluminium toxicity in plants can inhibits growth, uptake of nutrients and subsequently reduce crop yield whilst in humans it is associated with brain damage, nervous breakdown, memory loss, Alzheimers disease and gastrointestinal disorders (Jaishankar et al., 2014). However, zinc being an essential nutrient contributes to photosynthetic and growth processes, energy production and protein synthesis in plants (Tsonev and Lidon, 2012).

#### 3.2.3 Biological parameters

The results on the microbial characteristics of the different liquors and wastewaters are presented in Table 4. The means and the ranges are reported. The source of these microbial organisms (bacteria and helminth eggs) in the liquors could be from animal dung, dirt and manure trapped in the hairs/wool of the animal skins, process materials such as the plant parts (the pods and leaves are usually dried on the ground before use), or from erosion of contaminated soils during rainfall or by wind into the pits containing the liquors or wastewater. For the bacteriological analyses, the total coliform count ranged from 0–1.5 × 10^5^ in all the samples whilst the count for *E-coli* was zero. The levels of *E-coli* in all the wastewaters met the Ghana EPA permissible limit (10CFU/100mL) whiles the average count of the faecal coliforms exceeded the stipulated threshold limit (400 CFU/mL).

Their occurrence is a probable indication of environmental and faecal contamination of the liquors. It also depicts the presence of other pathogens in the liquors (Horan, 2003). The zero count recorded for *E-coli* and the coliforms may be attributed to the possible destruction of the bacterial cells by the high pH of the liming liquor as was observed by Grabow et al. (1978), the action of proteolytic enzymes (papain) in the pawpaw leaves used in the deliming/bating liquor, antimicrobial properties of the phenols (tannins, saponins and flavonoids) produced from the pods of the acacia and the sorghum leaf sheaths used in the vegetable tanning, black dye and red dye liquors (Ali Kauther Sir et al., 2020; Fayyad, 2014; Colak, 2006) as well as the high salt content, acidity and the excess aluminium ions in the alum tawing wastewater (Abdulkarim et al., 2009; Hajmeer et al., 2006; Bojić et al., 2002). Additionally, the high iron content of the black dye liquor might also play an inhibitory role against the growth of *E-coli* (Kalantari and Ghaffari, 2008).

Table 4 also shows the distribution of the helminth eggs in the different liquors. The eggs identified in the process liquors/wastewater were that of *Ascaris lumbricoides* (roundworms), hookworm (*Ancylostoma duodenale and Necator americanus), Trichuris trichiura* (whipworm)*, Strongyloides stercoralis* (threadworm) and *Enterobius vermicularis* (pinworm). These are human intestinal parasites typically found in impoverished areas of tropical and sub-tropical regions of the world. Eggs of *Ascaris lumbricoides* were the most predominant with the average count in all the liquors/wastewaters summing up to 16.60eggs/L which constitute 27.08% of the total number of eggs identified. Their high prevalence could be related to their abundance in the environment and also their ability to survive even under adverse environmental conditions (Dold and Holland, 2011). The second most abundant egg was that of the *Enterobius vermicularis* (15.70eggs/L; 25.61%) followed by *Trichuris truchuria* (10.10eggs/L; 16.48%), then *Strongyloides stercoralis* (9.8eggs/L; 15.99%), and then hookworm (9.10eggs/L; 14.85%).

Among all the liquors/wastewater, the vegetable tanning wastewater bore the highest number eggs (19.2eggs/L; 31.02%) whilst the alum tawing wastewater contained the least number. The average load of eggs in each of the spent liquors exceeded the limit set by WHO (2006) (<1egg/L) and therefore, cannot be considered for crop irrigation. The zero count recorded in the liquors could arise from the anthelminthic effect of phenols (contained in the plant materials used in the vegetable tanning, black dye and red dye liquors) (Ndjonka et al., 2014; Badar et al., 2011), high acidity (as in the alum tawing wastewater) (Hindiyeh and Al-Salem, 2004), papain, the proteolytic enzyme used in the deliming/bating liquor (Buttle et al., 2011; Stepek et al., 2005) and the high alkalinity and ammonia content of the liming liquor (Senecal et al., 2020; Jensen et al., 2009). Viable eggs and larvae of these worms are known to cause infection in man even at a low infective dose (Amoah et al., 2016). Infection may occur after penetration into the skin by an infective larvae or ingestion of the ova from contaminated hands, water, food or soil (Squire et al., 2018). The infection usually manifests itself in various diseases such as diarrhoea, undernutrition, abdominal bloating, kwashiorkor, stunted growth, lung and pelvis inflammation, low cognitive ability and anaemia particularly in pregnant women (Fan et al., 2019; Manz et al., 2017; Hossain and Bhuiyan, 2016). Thus, the tanners who practise poor personal hygiene and are inappropriately garbed in any protective clothing or equipment may be at risk of infection.

## 4 Conclusion

This study revealed that the amounts of water consumed and wastewater generated in the artisanal tannery are 1171 and 2120L/day and 820 and 1324L/day respectively. Much water is saved through the reuse of some of the process liquors. The concentrations of most of the contaminants in the artisanal tannery wastewater also exceeded the threshold limits set for wastewater discharge by the Ghana Environmental Protection Agency. High levels of COD, BOD_5_, turbidity, colour, TSS, TDS, chlorides, sodium, sulphates, total nitrogen and faecal coliforms detected in the wastewater could result in destruction of water bodies and toxicity in aquatic and terrestrial organisms. The count of the different helminth egg species in the spent liquors (*Ascaris lumbricoides, Ancylostoma duodenale, Necator americanus, Trichuris trichiura*, *Strongyloides stercoralis,* and *Enterobius vermicularis*) also exceeded the WHO guidelines for irrigation. Evidently, the indiscriminate discharge of the wastewater as practised by the tannery is a threat to human life and the environment. It is therefore, recommended that further studies should be carried out in development of efficient treatment systems to treat the wastewater to safe limit for either reuse or safe discharge. The tanners should also be educated on the use of personal protective equipment for their safety.

## Declarations

### Author contribution statement

Miriam Appiah-Brempong & Samuel Kwame Dadzie: Conceived and designed the experiments; Performed the experiments; Analyzed and interpreted the data; Wrote the paper.

Helen Michelle Korkor Essandoh, Nana Yaw Asiedu & Francis Warings Yao Momade: Conceived and designed the experiments; Wrote the paper.

### Funding statement

This work was supported by the Regional Water and Environmental Sanitation Centre Kumasi (RWESCK) at the 10.13039/501100012015Kwame Nkrumah University of Science and Technology, Kumasi with funding from Ghana Government through the World Bank under the Africa Centre’s of Excellence project.

### Data availability statement

Data included in article/supplementary material/referenced in article.

### Declaration of interests statement

The authors declare no conflict of interest.

### Additional information

No additional information is available for this paper.
